# Titanium as a Reconstruction and Implant Material in Dentistry: Advantages and Pitfalls

**DOI:** 10.3390/ma5091528

**Published:** 2012-08-24

**Authors:** Mutlu Özcan, Christoph Hämmerle

**Affiliations:** Center for Dental and Oral Medicine, Clinic for Fixed and Removable Prosthodontics and Dental Materials Science, University of Zurich, Plattenstrasse 11, Zurich 8032, Switzerland; E-Mail: christoph.hammerle@zzmk.uzh.ch

**Keywords:** biomechanics, corrosion, dentistry, implant, surface characteristics, titanium

## Abstract

Commercial pure titanium (cpTi) has been the material of choice in several disciplines of dentistry due to its biocompatibility, resistance to corrosion and mechanical properties. Despite a number of favorable characteristics, cpTi as a reconstruction and oral implant material has several shortcomings. This paper highlights current knowledge on material properties, passive oxidation film formation, corrosion, surface activation, cell interactions, biofilm development, allergy, casting and machining properties of cpTi for better understanding and potential improvement of this material for its clinical applications.

## 1. Introduction

Since the 1960s, titanium has become a popular metallic biomaterial because of its properties for many biomechanical applications including dentistry. Although there is an increasing trend for metal-free restorations in the dental profession, failures in the form of fracture or chipping associated with such materials are still being reported [1] indicating that there is still place for the indication of metal-ceramic fixed-dental-prosthesis (FDP). Furthermore, extensive oral rehabilitations could only be achieved with metal-ceramic FDPs since flexural strength of glassy matrix or oxide-based all-ceramic restorations do not allow for durable constructions of multiple-unit restorations [2]. On the other hand, the use of titanium as an implant material has become an integral part of dental therapy. The high cost of noble alloys and the potential biological hazards of the base metal alloys, were the main reasons for the introduction of commercially pure titanium (cpTi) and some of its alloys for the construction of dental prostheses and oral implants [3]. cpTi has been used in dentistry for more than five decades but there still seems to be potential to improve this material and its processing techniques in order to avoid possible biomechanical or biological complications. This paper will focus on current knowledge on cpTi and recent attempts to better understand and modify this material.

## 2. Material Properties of Titanium

Titanium is often used either as the pure metal, or in an alloyed form in aerospace applications, and in medical and dental work. It is commonly alloyed with other metals such as Vanadium (V) and Aluminum (Al). It forms then light-weight but at the same time strong alloys for the fabrication of oral implants or the frameworks for FDPs [2]. 

According to the American Society of Testing Materials (ASTM), cpTi is available in four different grades (Grade I-IV) that is based on the incorporation of small amounts of oxygen, nitrogen, hydrogen, iron and carbon during purification procedures, where each grade has different physical and mechanical properties. Grades I and II are the most commonly used cpTi types for the production of metal-ceramic FDPs [3]. In oral implants and implant-supported FDPs, cpTi and its alloys exhibit remarkable advantages due to their excellent biocompatibility, corrosion resistance, high strength, and low modulus of elasticity [3]. Yet, the recent trends in making dental biomaterials more biomimetic from both biomechanical and biological perspectives, also applied for cpTi.

Principally, the stress transfer between a metal framework and the dental tissues or bone is not homogeneous since stiffness (Young’s moduli) of the metal framework or implants and such tissues are different. This phenomenon is described as “stress shielding” [4]. In order to avoid devitalization of the tooth and atrophy in the bone under chewing function [5], high Young’s modulus of cpTi compared to those of the tooth and the bone structures, is not desirable. Young’s moduli of the most widely used SUS316L stainless steel and Co-Cr for orthopaedic implant devices, are approximately 180 GPa and 210 GPa, respectively [6] whereas Young’s moduli of cpTi and its alloys are generally lower than those of stainless steels and Co-Cr alloys. For example, cpTi and its alloy, Ti-6Al-4V, which is widely used for constructing implant devices, have a Young’s modulus of around 110 GPa. The growth of fibrous connective tissue on the implant surface is an important aspect to consider in order avoiding complications. Fibrosis can interfere with healing of the damage created during implant surgery [2]. In that respect, Ti-6Al-4V produce very little fibrous tissue and therefore bone can grow easily. However, this value still remains to be higher than that of the bone (10–30 GPa), enamel (80 GPa) and dentin (20 GPa) [7]. For this reason, attempts are being made to modify alloying cpTi without sacrificing from its mechanical properties.

cpTi alloys are generally classified as α-, (α + β)-, and β-type alloys. Young’s moduli of α- and (α + β)-type titanium alloys such as Ti and Ti-6Al-4V are higher than those of β-type titanium alloys. Generally, α-phase titanium is stronger but less ductile and β-phase titanium is more ductile. (α + β)-type titanium has mechanical properties which is in between both [5]. Therefore, β-type titanium alloys are considered more advantageous for the development of titanium alloys with low Young’s modulus for biomedical applications. Research lately has been focusing on reducing Young’s moduli of β-type titanium alloys for use in biomedical applications. A number of β-type titanium alloys, mainly composed of toxicity- and allergy-free elements and with low Young’s moduli have been developed over the years and are still being developed [8]. β-type titanium alloys have usually Young’s moduli of approximately below 80 GPa treated in special solutions [9]. Young’s modulus of a material can be different depending on the type of measurement methods used, such as tensile tests, three-point bending tests, and free resonance methods. The lowest value of Young’s modulus reported for the polycrystal β-type titanium alloy, Ti-35Nb-4Sn [10] or Ti-24Nb-4Zr-7.9Sn [11], subjected to severe cold working, is around 40 GPa. Among β-type titanium alloys, Ti-29Nb-13Ta-4.6Zr (Daido Steel Co., Ltd., Nagoya, Japan), referred to as TNTZ, has a low Young’s modulus of 60 GPa when subjected to solution treatment and measured by a resonance method [9]. This value was even decreased to around 55 GPa while keeping the tensile strength stable by severe cold rolling and cold swaging [8] such as high pressure torsion, accumulative roll-bonding, and equal channel angular pressing [12]. Improving static strength such as tensile strength can be achieved by employing strengthening mechanisms by means of work hardening, grain refinement strengthening, precipitation strengthening, and dispersion strengthening [8]. 

The strength as well as Young’s modulus of titanium alloys is a very important material property for their long-term durability when used as implants for biomedical applications. In particular, dynamic strength under fatigue conditions is highly important. Increasing the fatigue strength and simultaneously decreasing Young’s modulus is somewhat difficult because they are opposite natures when the forces between atomic arrangements are considered [5]. The fatigue strength of cpTi is significantly improved over the years by conducting aging treatment after solution treatment or thermo-mechanical processing including severe cold working followed by aging treatment. The addition of small amount of ceramic particles in the matrix is also expected to improve the fatigue strength of β-type titanium alloys while maintaining low Young’s modulus. Young’s modulus of TNTZ, for instance with Y_2_O_3_ addition, subjected to severe cold rolling is nearly constant at around 60 GPa and it increases as a function of increased Y concentration. Relationships between tensile strength and elongation of TNTZ added with different amounts of Y_2_O_3_ addition, subjected to severe cold rolling showed excellent balance [13]. 

The effect of Young’s modulus on bone atrophy and bone remodeling has been widely investigated using implants made of titanium alloys with different Young’s moduli [14]. The challenge remains as the simultaneous improvement of the dynamic strength and lowering Young’s modulus of the β-type titanium alloy, TNTZ. Of course, not only the material property as such but also the geometry of the reconstruction plays a major role to control Young’s modulus. Fortunately, as an oral implant material, clinical studies demonstrated limited failures in the implant material itself [15]. cpTi as an FDP framework material veneered with ceramics has not been evaluated long term in clinical studies. However, in general metal failures, regardless of the type of metal framework, perhaps with the exception of extreme bruxism cases, is a rarely experienced failure type in dentistry. Nevertheless, stress shielding between the reconstruction and the dental tissues, the biological response of the dental tissues and bone needs to be proved especially with new-alloyed titanium materials.

## 3. Passive Surface Oxide Film on Titanium

Corrosion processes cause a reaction film to form on metallic materials. Certain metals like Ti oxidize easily, forming a very thin, stable passive layer that is self-limiting and protects the surface of the metal from further oxidation. Due to the tremendously fast rate at which they are formed, passive films, which are 1 to 5 nm thick, readily become amorphous [16]. The surface of oxide reacts with moisture in air and hydroxyl groups are rapidly formed within 30 ms. In the case of Ti, the surface oxide immediately reacts not only with water molecules in aqueous solutions but also with moisture in air and is covered by hydroxyl groups [16]. The surface hydroxyl groups contain both terminal OH and bridge OH in equal amounts. The surface oxide contains a hydroxide or hydroxyl group (OH) and water. This behavior, passivity, gives Ti its high corrosion resistance under certain controlled conditions where, otherwise, it would undergo strong active corrosion. The passive oxide layer formed on the surface of Ti is also considered responsible for its good biological performance, as it is less reactive than bare Ti [17]. Since amorphous films hardly contain grain boundaries or structural defects, they are usually corrosion resistant. However, corrosion resistance decreases with crystallization [17].

Metals can have stable passivity, where the oxide layer self-heals immediately after being ruptured, or they may present unstable passivity, where the oxide layer is unable to heal after disruption and the bare metal is exposed to active corrosion [18]. Both of these events depend on the oxidizing or reducing potential of the environment. On the other hand, active surface hydroxyl groups dissociate in aqueous solutions and form electric charges. Positive or negative charge due to the dissociation is governed by the pH of the surrounding aqueous solution where the positive and negative charges are balanced and the apparent charge is zero at a certain pH. This pH is the point of zero charge (pzc) [18]. The pzc is the unique value for an oxide and an indicator that the oxide shows acidic or basic properties. For example, in the case of TiO_2_, the pzc of rutile is 5.3 and that of anatase is 6.2 [18]. In other words, the anatase surface is acidic at lower pH and basic at higher pH than 6.2. Active surface hydroxyl groups and electric charges formed by the dissociation of the groups play important roles in the bonding with polymers and immobilization of molecules.

## 4. Corrosion and Clinical Relevance

Most materials chosen for prosthetic applications exhibit passivity properties and, thus, relatively low corrosion rates compared with those of other more reactive metals, such as Zinc, Magnesium, or Vanadium, which undergo active corrosion even in relatively neutral pH [19]. In the oral environment, extreme acidic conditions do not exist but the constant aqueous environment coupled with the biofilm effect, fatigue forces and possible interaction with other metals in the mouth may impair the passive surface oxide film [19]. Such environmental conditions can breach the protective oxide layer formed on the surfaces of these passive materials and cause corrosion, affecting the mechanical integrity of the implant and the health of the surrounding tissue. Extreme acidic conditions found during inflammation [19], fretting between implant and bone [20], and galvanic corrosion between Ti implants and other metallic alloys used for common dental procedures could greatly affect the mechanical stability and clinical outcome [21]. Eventually, implant surface properties such as roughness, chemistry, and energy directly influences tissue response by affecting protein adsorption and modulating cell proliferation and differentiation [22]. Additionally, innovations in surface modification techniques have improved the biological performance of metallic implants [23,24]. However, some modifications may diminish mechanical properties of the bulk material, resulting in surface micro-cracks, increased corrosion rates [25,26], and, thus, increased corrosion currents that may affect surrounding cells and tissues.

Corrosion of metallic implants, a topic extensively discussed in orthopedic literature, may jeopardize the mechanical stability of the implant and the integrity of the surrounding tissue [27,28]. Implant failure in the form of aseptic loosening, or osteolysis, may result from metal release in the form of wear debris or electrochemical products generated during corrosion events [29,30]. Metal ions such as Ti^4+^, Co^2+^, and Al^3+^ have been shown to decrease DNA synthesis, mitochondrial dehydrogenase activity, mineralization, and mRNA expression of alkaline phosphatase and osteocalcin in ROS 17/2.8 cells [31]. Similarly, phagocytosis of Ti particles caused cytotoxicity in a concentration-dependent manner in rat calvarial osteoblasts and MG63 cells [32,33]. 

While implant loosening is less prominent in the dental literature, metal traces originating from dental implants have been found in blood, liver, lungs, and lymph nodes [34,35,36]. These metal ions and wear debris may also contribute to aseptic loosening by promoting inflammatory complications that may result in macrophage activation, bone resorption, and, rarely, in the potential development of neoplasia [37]. In 2006, titanium dioxide (TiO_2_) was classified as possibly carcinogenic to human beings (*i.e.*, group 2B) at the International Agency for Research on Cancer (IARC) [38]. Animal studies in rodents provided sufficient evidence of the carcinogenic effects of TiO_2_, although epidemiological cohort studies in humans were inconclusive. Furthermore, the immediate and systemic cytotoxic and neoplastic effects of corrosion remain controversial because of conflicting studies that have found no effects of Ti ions or Ti particles on cells [39]. Moreover, the nanograms of metal per gram of tissue found *in vivo* [40] are difficult to compare with the micrograms and milligrams of metal per milliliters of solution used to create an effect in *in vitro* studies [32,33]. 

The electrical implications of corrosion and its effect on the surrounding tissue may be an important aspect to focus in future studies, but such effects still remain unclear. Corrosion events generate electrical currents due to electron transfer from ions in the solution or to the metallic surface where reactions are occurring. These abnormal currents, and coupled electrical potentials, are directly related to the cyclic loads applied to the implant [28]. As described previously, bone cells are sensitive to electrical signals and, thus, could be strongly affected by these corrosion currents [19]. Moreover, these abnormal electrical signals may provide an alternate explanation for the unresolved causes of inflammatory complications and eventual aseptic loosening. 

With the growing popularity of treatments like early implant loading, it is imperative to consider the effects of electrical signals on the early stages of osseointegration as well as on long-term outcome. The concern of reducing implant corrosion might be addressed by new formulations of metallic alloys that improve the mechanical and corrosion properties of the implant [41,42], surface modifications that stabilize the reactivity of the surface [25] or electrical protection of implants. In particular, clinical implant failures, be it due to peri-implantitis or other reasons, need closer investigation of the material surface properties.

## 5. Titanium Surface Activation

The use of bone-anchored titanium implants has become routine treatment modalities in dentistry. Apart from surgical procedures, the success of implants is highly dictated by the surface properties of the implant material that influence molecular interactions, cellular response and thereby, bone regeneration. Mesenchymal stem cell involvement, cell-cell communication at the bone-implant interfaces and in particular interactions between the surface oxide and the biological host are the underlying mechanisms of osseointegration. 

Titanium implants, machined to a smooth surface texture, has been used more than 50 years clinically. Over the years chemical and topographical modifications moderately changed surface topography. Evidence from *in vitro* and *in vivo* studies suggests that surface modifications on titanium implants promote a more rapid bone formation than do machined surfaces [43]. Ultrastructural modifications such as using ultrathin calcium phosphate coatings provide bonding on the atomic scale between the oxide and apatite nanocrystals [44]. Chemical and biological interactions between the coated titanium surface and the host tissue start with binding of water molecules, ions and biomolecules, followed by mineralization at the implant surface. Therefore, the initial state of the titanium surface is decisive for the tissue regeneration around the implant. 

Surface topography and surface roughness of oral implants have been modified from micro- to nanometer scales, which are partially dependent on the oxide or the bulk material. It is generally accepted that surface roughness on the micrometer scale plays an important role for cellular reactions, tissue healing and implant stability [45]. Different methods such as machining, air-abrasion, acid etching, electrochemical oxidation and laser treatment are used to alter surface topographies on titanium implant surfaces at various thicknesses. 

In anoxic acid media, complete dissolution of anodic oxide films on titanium can occur, leading to rapid corrosion [46]. Furthermore, these films on titanium are highly doped n-type semiconductors that grow via a high field growth mechanism and tend to be amorphous. The donors are believed to be Ti^3−^ ions trapped in the process of migrating to the oxide-electrolyte interface, with the level of the donor density decreasing with increasing film formation rate. To circumvent these problems, typically the titanium implant surfaces are air-abraded with aluminum trioxide particles [47]. Blasted implants demonstrate better bone integration than turned/machined implants. In addition, blasted surface are also etched in some products. Different Ra/Sa values reported in the studies may be a result of different measurement equipments and evaluation techniques. In contrast to animal studies, clinical studies often fail to find any major advantages or disadvantages with blasted implants when compared with turned implants [47]. Nevertheless, after air abrasion, implant surfaces are coated with calcium phosphate/hydroxyapatite (HA). Initially, it was applied with plasma spraying by melting the coating material and spraying it onto the titanium surface. Early experience with these coatings suffered from adhesive debonding from the titanium surface or cracking. Later, evaporation coating such as physical vapor deposition (PVD) was tried where HA was sputtered onto the surface layer by layer that presented adequate adhesion on the titanium surface [48]. One limitation of this kind of coating is the surface geometry. Although it works ideally on flat surfaces, complex geometries could not be coated evenly using the PVD method. HA coatings were then tried to be applied using wet chemical processes, such as sol-gel technologies [49,50]. Although biological impact of such coatings is not well-established, parameters such as immersion time, temperature, may affect the thickness, morphology and composition of the coatings. 

Covalently bonded, self-assembled monolayers such as silane coatings and thiols with different functional groups (*i.e.*, methyl, hydroxyl or carboxyl groups) are also being tried to modulate the hydrophilicity of the titanium surface [51]. Peptide sequences have been used as monolayer modifications and shown to promote cell adhesion [52]. Recent attempts for activating titanium surface try to incorporate local release of pharmacological substances from the implant or graft material. One example is the use of bisphosphonates that downregulate osteoclast activity and result in more rapid bone regeneration around implants [53,54]. Although the clinical implication of monolayer modified implants remains to be verified, target oriented micro-, nano- or meso-scale features incorporating proteins or other biologically active substances seem to constitute future activation methods on titanium oral implants. 

## 6. Titanium-Cell Interactions

Bone regeneration around oral titanium implants goes through stages of inflammation, regeneration and remodeling with possible overlap at all these phases. In the absence or presence of titanium implants both healing types show variations on the cellular and molecular level. In the presence of an implant, the implant itself acts as an osteoconductive substrate decreasing the size of the defect to be bridged by the new tissues. The titanium implant surface influences the initial sequences of protein adsorption, platelet adhesion, haemostasis, inflammation and osteogenic cell response [55,56,57]. In that respect, physico-chemical properties of the implant surface interacting with the cellular pheno- and genotypes at the immediate vicinity of the surface, molecular mechanism of how and in which sequence the cells are recruited and become adherent to the surface has to be defined. Moreover, the cell-cell communication during the early phase of osseointegration requires better understanding. 

Immunohistochemistry and SEM analysis show gene expression of cells adherent to titanium implants during the first hours and days after implantation [58,59] with higher expression of monocyte chemoattractant protein-1 (MCP-1) coupled with higher expression of pro-inflammatory cytokines TNF-a (3 h and 1 day after implantation) and IL-1b (1 day and 6 days after implantation) at machined implant surfaces. On the other hand, the expression of the chemokine receptor CXCR4, a receptor for stromal derived factor-1a (SDF-1a), was highly expressed at oxidized surfaces as early as 12 h after implantation. Coexistence of monocytes/macrophages and mesenchymal stem cells (MSCs) at the interfacial region with predominance of MSCs at the oxidized surface has been identified. It has been also shown that early peak expression of SDF-1a during the first day after tissue injury was associated with the highest MSCs at the injury site [60]. The role of SDF-1a/CXCR4 chemotactic axis in mediating the recruitment of progenitor cells is of current research interest to reveal the mesenchymal cell recruitment to different sites of healing [61]. 

Cell attachment to the implant surface is one of the critical first steps in the cell response to a biomaterial [56]. The cellular attachment is mediated through a protein-rich layer through adhesion receptors including the integrins. Cells adherent to oxidized surfaces showed upregulation of integrin b1 during the 24 h of implantation [58,59]. In addition to osteogenic cells, monocytes and other cells also express integrin b1. Moreover, integrin b2, expressed mainly by leukocytes, was higher at the oxidized implants after 12 h of implantation [61]. This integrin is also expressed by osteoclast progenitors [61].

Inflammation at the bone-implant interface has not received much attention as that given to the soft tissue-implant interface. Histological studies in bone revealed that macrophages and multinucleated cells are present in machined titanium implants [62] as well as HA-coated implants [63] during the early stage after implantation. These cells are known to express a wide range of pro-inflammatory and anti-inflammatory cytokines, growth and differentiation factors and chemotactic mediators. Major pro-inflammatory cytokine, TNF-a, was upregulated after 3 h at the machined implants compared with oxidized ones [58]. Higher expression was also observed at that surface after 1 day. Down regulatory effect was observed on the expression of IL-6 at titanium implants blasted with TiO_2_ particles and subsequently treated with hydrofluoric acid [64]. On the other hand, this surface was associated with higher expression of anti-inflammatory cytokine IL-10, eight weeks after implantation in rabbit cortical bone. Since many cell types, including osteoblasts, can express these cytokines and growth factors it is still important to define which cell type is responsible for these changes in gene expression. For instance, the expression of IGF-1 was also upregulated at HF surface during the eight-week evaluation period. *In vitro* studies have demonstrated that monocyte cell line expressed BMP-2 [65] that contributed principally to the osteogenic differentiation. However, *in vivo* data is not available showing the expression of BMP-2 from monocytes during osseointegration. Research based on antibody-labelling strategies such as immunohistochemistry and fluorescence assisted cell sorting are suggested to verify these findings [66]. 

The regulation of gene expression at implant surfaces clinically is a complex phenomenon. The material properties possibly influence the gene expression by affecting transcription factor, such as RUNX2, in the differentiation of mesenchymal cells towards the osteoblastic lineage. This factor has also been shown to contribute to the osteoclastic differentiation [67]. The higher expression of osteoblast markers alkaline phosphatase (ALP) and osteocalcin (OC) and osteoclast marker cathepsin K (CATK) was parallel to a higher expression of RUNX2 at the oxidized surfaces compared with machined ones after 3 days of implantation [59]. Similar results were demonstrated for HF surface in comparison to surfaces without acid etching [59]. Although all of these studies suggest fast and strong influence of the different material surface properties on the expression of critical switching factors, it is still not revealed in which way and which specific surface properties contribute to such effects. 

Recent studies on early osseointegration (hours-days) have demonstrated that the upregulation of genes responsible for bone formation ALP and OC was coupled with upregulation of genes expressed by osteoclasts indicating that the bone remodeling phase is triggered much earlier than what has previously been assumed [59]. An intimate communication is established between osteogenic cells and osteoclasts. For instance, the surface receptor RANK on osteoclasts recognizes and binds to osteoblast membrane-associated factor (RANKL) during the osteoclastic differentiation from the monocytic lineage [68]. Briefly, active bone resorption and bone formation takes place starting already during the first days after implantation and continuing over time. Studies evaluating the gene expression of interfacial cells in combination with other surface properties of the implants are needed in order to extrapolate possible changes at implant-tissue interface, taking also coating or oxide stability on titanium surface into account.

## 7. Titanium-Biofilm Interactions

The implant surface is susceptible to infection because of two main reasons, namely formation of a surface biofilm and compromised immune ability at the implant/tissue interface. The biocompatibility of titanium implant can be attributed to a surface protein layer formed under physiological conditions [69]. This protein layer actually makes the surface suitable for bacterial colonization and biofilm formation [70,71,72]. Biofilms are defined as a microbially derived sessile community characterized by cells irreversibly attached to a substratum, interface or to each other, embedded in a matrix of extracellular polymeric substances that they have produced, and exhibiting an altered phenotype with respect to growth rate and gene transcription [73]. Concerning the pivotal role that biofilm plays in implant-associated infections, the process of biofilm formation has been well documented [71,72,73]. The biofilm protects adherent bacteria from the host defense system and bactericidal agents via several proposed mechanisms [74]. The host immunity ability on the implant is consequently impaired. In the early phase after implantation, the local defense system is severely disturbed by the surgical trauma, and so it is the most dangerous time for infection. Even after completion of tissue integration, the defense ability at the implant/tissue interface is still compromised on account of the small number of blood vessels in this zone [69]. The reduced defense mechanism facilitates colonization of bacteria and infection may result. Although various measures such as thorough disinfection and stringent aseptic surgical protocols have been proposed to mitigate bacterial contamination, there is still evidence that bacterial invasion usually occurs after surgery [75]. Bacterial contamination can also arise from hematogenous sources at a later time [76]. Measurement of the water contact angle shows a nominal increase in the hydrophilic nature of TiO_2_ films, whereas the surface energy increases with decreasing contact angle [69]. Bacterial species Staphylococcus aureus and Escherichia coli interaction with nanostructured surfaces showed an increase in adhesion and biofilm formation with increasing nanoscale morphological properties [69]. 

Research in this field tried using chlorhexidine that can adsorb to the TiO_2_ layer on the titanium surface and desorb gradually over a period of several days [77,78]. Several kinds of coatings fabricated on titanium with and without chlorhexidine adsorption were studied [79,80]. The chlorhexidine release pattern is similar to that of antibiotic coatings with an initially rapid release rate followed by a period of slower but sustained release [79]. Chlorhexidine can also adsorb onto the titanium implant surface modified by the covalent coupling of collagen on a polyanionic acrylic acid overlayer via the ionic interaction between the cationic chlorhexidine and polyanionic collagen surface [81]. Other alternative methods have also been employed to form coatings comprising non-antibiotic organic antimicrobial agents on titanium [82]. 

More recent studies are trying to tune the TiO_2_ by using novel facile nanosphere colloidal lithographic technique in combination with biomolecular patterning [83,84]. 

## 8. Titanium Allergy

It should be noted that no material can be considered universally biocompatible and this does not exclude titanium [85]. It is recognized that environmental factors are contributing to the increase in the frequency of allergic disorders affecting world populations [86]. Dental biomaterials release substances that alter the oral environment to a varying degree [87,88] and thus may contribute to local allergic reactions within the oral tissues. In the oral cavity, an elevated concentration of metal ions may be harmful and act as a local immuno-suppressant [89]. Recently, it has been suggested that titanium hypersensitivity may be a factor responsible for implant failure [90,91,92]. Although titanium hypersensitivity is a growing concern, epidemiological data on the incidence of titanium-related hypersensitivity reactions are still lacking. 

Degradation products of metallic biomaterials including titanium may mediate metal hypersensitivity or allergic reactions [93]. Titanium and other elements released from titanium implants have been observed in tissues and organs near implants [94]. The cause of ion release other than wear and fretting from titanium is unclear. Released titanium debris or ions may combine with biomolecules such as native proteins or form a protein-metal complex and become immunogenic, eliciting a Type-IV T-cell mediated response [95]. Even though titanium has been regarded as an inert metal, several earlier studies have identified potential hematologic and metabolic toxicity [85]. Reports relating to titanium toxicity are sparse but concur that cationic titanium and soluble titanates are relatively non-toxic in the amounts and forms that are normally ingested, due to poor absorption from the mammalian alimentary tract [85]. Furthermore, it seems possible that the incidence of allergic reaction to titanium implants may be under-reported due to a lack of recognition as a possible etiological factor in implant failure. Long-term clinical and radiographic follow-up of patients who have had an implant and who are diagnosed with metal sensitivity needs to be documented. At present, little is known on titanium hypersensitivity, but it cannot be excluded as a reason for implant or reconstruction failure. 

## 9. Dental Titanium Casting

Despite the favorable characteristics, cpTi casting for prosthetic purposes has not been viable for many years since casting procedures led to the formation of an undesirable crust resulting in high reactivity and fragility [96]. This coating is called “alpha-case” and is formed by incorporation of the elements from the investment that may impair the adhesion between cpTi and ceramics for FDPs [97]. In order to allow the utilization of titanium, specific equipments were developed for casting, refractory investments were manufactured that were different from the conventional silica-phosphate investments, and ultra-low temperature ceramics were produced to be able to fire them on cast cpTi [98]. Only then cpTi was indicated for the fabrication of removable and FPDs. 

Dental castings are made via pressure-vacuum or centrifugal casting methods [99]. The metal is melted using an electric plasma arc or inductive heating in a melting chamber filled with inert gas or held in a vacuum. The molten metal is then transferred to the refractory mold via centrifugal or pressure-vacuum filling. Casting technology using a centrifugal casting machine with induction heating housed in a vacuum chamber to cast titanium implants. It was not until around 1977, that experimental castings of titanium for crowns and partial denture frames were finally made by induction melting in vacuum. It was difficult to maintain consistency in titanium dental castings because of their inherently poor castability. Though titanium is economical, biocompatible and readily available, technologies for easier casting, machining, welding and veneering are still necessary. Oxidation during melting and casting and the reaction of the molten metal to the investment material should be minimal. With the advancements in casting technologies, the alfa-case layer was controlled better by induction in an environment with inert atmosphere of argon or helium gas and with the use of refractory investments that contain oxides such as magnesium, yttrium and zirconium [100,101,102]. 

The control of alfa-case layer still remains a problem. Kimura *et al.* [103] identified the presence of four layers after cpTi casting and the authors advocated removal of the two first layers before application of the ceramic layer. Elimination of the superficial layer is however performed by utilization of a carbide burs at low speed. Since this procedure is operator driven by the dental technicians in the laboratories, it can never be made sure that alfa-case layer is removed in a controlled fashion. The oxide layer formed on the surface of the melted cpTi alloys presents great chemical stability. However this layer, at the same time, could impair chemical reactions between the cpTi and the bonder ceramic components, weakening the cpTi-ceramic union. Moreover, oxide layer is often not adhered sufficient, present a porous form and therefore it has been previously shown that oxide layer itself or the interface between the oxide layer and the alloy was responsible for the metal-ceramic fractures [103,104,105]. Recently, the mean flexural strength values for the ceramic-gold alloy combination were found significantly higher than those of the ceramic-cpTi combination regardless of the fatigue conditions performed [106]. Microscopic analysis of the specimens after flexural strength test showed complete adhesive detachment of the ceramic from the cpTi frameworks exclusively, indicating the weakest interface of the assembly was still located between the cpTi framework and its oxide layer. For these reasons, other processing routes are being developed for cpTi. To date, however, there is limited clinical data available that compare the long-term success of titanium restorations with those made from more traditional metals [107,108]. Hence, the severity of the problem remains scarce.

## 10. Dental Titanium Machining

Dental implants generally are machined from billet stock of pure metal or alloy. Dental crowns and bridge frameworks are also possible to be machined from solid metal stock via computer-aided machining (CAM) [109]. Abrasive machining of titanium, however, is slow and inefficient, which greatly limits this approach. Another method for fabricating dental appliances is electric discharge machining, which uses a fabricated graphite die, often reproduced from the dental working die, to erode the metal to final shape via spark erosion [110]. Multiple dental prostheses can be machined using CAM systems but implementation of such devices is very expensive. For these reasons, the use of titanium for dental castings has not become a prevalent laboratory and clinical practice. Currently, to eliminate possible limitations in casting or machining, materials with low reactivity are used to prevent surface reaction with the molten metal, and materials with high setting expansion are used to compensate for the high casting shrinkage of titanium. Future CAM procedures are needed for easy handling and controlled oxide formation on the cpTi.

## 11. Conclusions

Both titanium and titanium alloys, based on their physical, chemical and biological properties, appear to be especially suitable for dental implants and prostheses. For the construction of endosseous implant devices, titanium and its alloys have become well-accepted and can be considered the materials of choice. Surface activation or tuning of titanium surfaces certainly will improve biological integrity in compromised situations, increasing clinical service of implant therapies even further. Processing difficulties, however, have limited usefulness of titanium in fixed and removable prostheses in dentistry. For crown and bridge prostheses, dentists can consider titanium and its alloys as viable options to more traditional noble and base metal alloys, but careful selection of processing methods and laboratory skill are necessary to ensure success. 
